# The Role of Promoting Agricultural and Food Products Certified with European Union Quality Schemes

**DOI:** 10.3390/foods13060970

**Published:** 2024-03-21

**Authors:** Alexandra-Ioana Glogovețan, Cristina Bianca Pocol

**Affiliations:** Department of Animal Production and Food Safety, University of Agricultural Sciences and Veterinary Medicine of Cluj-Napoca, 400372 Cluj-Napoca, Romania; alexandra.glogovetan@gmail.com

**Keywords:** protected designation of origin (PDO), protected geographical indication (PGI), traditional specialty guaranteed (TSG), Romanian consumers, cluster analysis, segmentation, European Union (EU) quality schemes, geographical indications (GI)

## Abstract

This study delves into the effectiveness of digital marketing strategies in promoting local agricultural and food products in Romania, certified with European Union quality schemes. By examining consumer profiles, preferences, and their awareness of EU quality labels, the research uncovers the motivations driving purchasing decisions and the influence of digital marketing on these choices. Utilizing quantitative methods, including a comprehensive survey across 903 respondents, the study identifies four distinct consumer segments: Eco−Advocates, Les Connaisseurs, Price−Sensitives, and Traditionalists. These segments exhibit unique behaviors and attitudes toward certified products. The research highlights the potential of digital marketing to significantly alter consumer behavior toward EU-certified products, underscoring the importance of tailored communication strategies. It contributes to the understanding of consumer segmentation in the context of European Union quality schemes, providing valuable insights for policymakers, marketers, and producers. The findings advocate for enhanced digital marketing efforts to increase awareness and appreciation of these certified products, thereby supporting the broader objectives of quality and certification in the European agricultural and food product sector.

## 1. Introduction

The European Union’s (EU) quality policy for agricultural and food products represents a cornerstone in the Union’s efforts to preserve unique regional food cultures while simultaneously promoting agricultural diversity and consumer trust [1]. This policy framework encompasses several key certification schemes, each aimed at safeguarding and highlighting distinct product characteristics tied to geographical origin and traditional practices. Among these, the Protected Designation of Origin (PDO), Protected Geographical Indication (PGI), and Traditional Specialty Guaranteed (TSG) are the most notable. PDO certification is reserved for products that possess a unique quality or traits specifically linked to their geographic setting, encompassing both natural elements and human influences. This designation implies that all stages of the production process—cultivation, processing, and crafting—occur within the specific region, ensuring that the product’s qualities are inherently linked to its place of origin. Such a stringent link between product and place not only preserves traditional methods but also enhances the product’s reputation and market value [1].

The PGI label is slightly less restrictive than PDO, requiring that at a minimum, one aspect of production, processing, or crafting stages occurs in the designated area. This certification highlights the connection between the distinct geographic area and the product’s name, wherein a unique quality, reputation, or distinct feature is fundamentally linked to its place of origin. The PGI status aids in promoting regional products on a broader scale, allowing for a more inclusive approach to quality certification [1]. Unlike PDO and PGI, the TSG certification does not link the product to a specific geographical area but rather focuses on traditional composition or means of production. This designation is key in preserving traditional recipes or methods, which are a part of the traditional and gastronomic heritage. TSG helps in maintaining a high standard of authenticity and contributes significantly to the diverse culinary landscape of the EU [1].

These certifications serve to safeguard the names of particular products to enhance their distinctive traits, associated with both their geographic origins and conventional expertise. This policy framework plays a crucial role in maintaining rural economies by supporting local producers and preserving the environment. It also contributes to consumer protection by ensuring quality and authenticity [2]. Moreover, these labels are instrumental in guiding consumer choices, providing assurance regarding the quality and provenance of the products. The implementation of these quality schemes by the EU underscores a commitment to maintaining a high standard of food quality, regional authenticity, and consumer transparency [3]. As the global market expands and consumer preferences evolve, these certifications have become pivotal in distinguishing the uniqueness of European agricultural and food products. They represent the blend of tradition, quality, and diversity that characterizes the agricultural heritage of the EU. Consequently, understanding the impact and significance of these labels is not only essential for regional producers and the agricultural sector but also for consumers who increasingly value authenticity and quality in their food choices [4].

The current landscape of consumer behavior research highlights a significant gap in understanding the practical applicability of consumer segmentation into clusters, especially concerning food products with European quality schemes (PDO, PGI, TSG). This gap is not merely academic but has real-world implications, particularly for policymaking and market strategies. Although consumer awareness of EU quality labels on agricultural and food products is gradually increasing, studies have shown that this awareness is still relatively low to medium overall [5,6,7]. Awareness tends to be higher among certain demographics, such as frequent Internet users, those in specific occupations, and wealthier households [8]. However, there is a significant portion of the consumer base that remains less informed about these labels and their implications [9].

This gap in understanding consumer segmentation and the nuances of consumer behavior significantly hinders the development of targeted marketing strategies and an enhanced comprehension of consumer inclinations and buying patterns. While the EU’s quality schemes provide several benefits to producers, including fair competition and a return for farmers, the main limitations lie in the low consumer awareness and understanding of these schemes in some EU countries [10]. Addressing this knowledge gap is crucial for policymakers and marketers alike, as a more comprehensive understanding of consumer segments can lead to more effective marketing campaigns and policy decisions by resonating with consumer needs and preferences in the context of EU quality scheme agricultural and food products.

Thus, the aim of this research article is to evaluate the influence of digital marketing on the behavior of Romanian consumers in choosing Romanian agricultural and food products certified with EU quality schemes. The research objectives were as follows:

O1. Identifying the profile of the consumer of agricultural and food products certified with EU quality schemes and their preferences based on age, gender, quality/price ratio, and level of education.

O2. Identifying consumers’ knowledge about agricultural and food products certified with EU quality schemes.

O3. Identifying the reasons for purchasing and consuming agri-food products certified with quality schemes.

This research article is structured to provide a comprehensive analysis of consumer behavior in relation to agricultural and food products certified with EU quality schemes. It follows with a literature review by synthesizing existing knowledge. This is succeeded through an in-depth explanation of the methods used, encompassing the research design, data gathering, and analysis methods. The results and discussion sections present the findings and interpret their implications. The article concludes with recommendations based on the study’s insights, a summary of its limitations, and suggestions for future research directions in this field.

## 2. Literature Review

### 2.1. EU Quality Schemes: Context, Advantages, and Challenges

The PDO, PGI, and TSG designations have been designed to protect and promote products with distinctive traits associated with their place of origin and traditional know-how. These schemes confer intellectual property protections to specific agricultural and food products, enhancing consumer trust and enabling producers to better market their products. The recognition of a product as PDO, PGI, or TSG enables consumers to distinguish quality products, which, in turn, aids producers in achieving a fair return and competitive edge in the market [11]. The PDO certification, for instance, requires that the entire production, processing, and crafting process must occur within a designated area. This strong link to the place of origin ensures the authenticity and uniqueness of the product [11]. For instance, Kalamata olive oil PDO is exclusively produced in the Kalamata region of Greece, utilizing types of olive native to that locality [12]. On the other hand, PGI places less emphasis on the geographical link, requiring that at least one of the production stages occurs in the region. This flexibility allows for a wider range of products to be included under this scheme, such as the Westfälischer Knochenschinken PGI ham from Germany [13]. TSG focuses on protecting traditional aspects of production or composition, irrespective of the geographical area. This scheme is instrumental in preserving traditional methods and recipes, ensuring their continuation and protection against falsification and misuse. An example of a TSG product is Gueuze, a traditional Belgian beer known for its spontaneous fermentation method [14]. Consumer trust holds a pivotal position in the achievement of such certified products. Studies [10,15] have shown that consumers have a favorable disposition toward PDO labels due to the guarantee of quality and support of the local economy these labels signify. However, the low and fragmented recognition of these labels among consumers can jeopardize their effectiveness. This highlights the need for increased consumer awareness and education to ensure that the benefits of these labels are fully understood and appreciated [15].

Despite the advantages brought by these EU quality certifications, there are inherent risks and challenges associated with them. The perception of foods by consumers is greatly influenced by authenticity and origin, and food fraud significantly affects this perception [16]. Understanding consumer attitudes toward food fraud and authenticity is crucial for developing effective food fraud prevention strategies. It is essential for the industry and governments to prioritize communication and mitigatory actions regarding food fraud, particularly concerning PDO, PGI, and TSG agricultural and food products [17].

One of the principal risks is the low consumer awareness and understanding in some EU member states, which can hinder the effectiveness of these labels in driving consumer choice. Additionally, complex and lengthy registration procedures can be a deterrent for producers, particularly in the case of TSG, where the perceived low added value for registration due to low consumer awareness has been identified as a significant challenge [10]. Moreover, while these schemes are relevant and coherent with EU policies and trademarks, the challenge lies in effectively communicating the benefits and distinct qualities of these certified products to consumers. Enhancing consumer education about the significance of these labels and their distinction from conventional products is critical for maximizing their impact [18].

Another risk is the food fraud in the realm of PDO, PGI, and TSG food products, which represents a significant economic burden, estimated to cost between USD 10 to 65 billion annually globally. Dairy products, especially cheeses with protected labels, are among the most impacted by food fraud, as consumers are willing to pay a higher price for these supposedly authentic products [19]. This fraudulent activity includes mislabeling, use of non-authentic ingredients, and misleading marketing to exploit the higher market value of certified products. The implications of such fraudulent activities are extensive, impacting both consumer health and producer economics. Consumers face health risks from products that may not meet safety standards, while producers suffer economic losses and damage to the reputation of genuine certified food products [19,20].

To combat food fraud, advancements in food fraud detection methods have surfaced as significant instruments for food verification. These methods are increasingly being developed and employed to ensure the authenticity of PDO, PGI, and TSG food products, particularly in the domain of probiotic items and fermented foods and drinks [21]. Other advancements in technology, such as a stable isotope ratio analysis, are proving to be valuable tools in verifying the authenticity of PDO, PGI, and TSG food products. These technologies help in accurately determining the origin and ingredients of products, thereby aiding in the fight against food fraud. Such technological interventions are crucial in maintaining the integrity of these certifications and ensuring consumer trust [21,22]. Thus, effective regulation and consumer education are key in combating food fraud. Agricultural and food fraud is combated via measures such as quality checks, supervision of control bodies, and investigations, especially in the realm of PDO and PGI products. Increasing consumer awareness about food fraud and the importance of these certifications is essential, as many consumers are only partially familiar with these issues, so building consumer trust not only requires a concerted effort thread from regulatory bodies and producers, but also from marketers [23,24].

### 2.2. Consumer Trends and Certification

The evolution of food quality perception among consumers has significantly influenced the demand for EU-certified food products. Historically, food was deemed high quality based on the absence of defects or adulteration [25]. However, contemporary definitions of quality encompass both intrinsic qualities, such as color, taste, and aroma, and extrinsic characteristics like environmental impact and origin. EU quality schemes, by emphasizing these qualities, play a pivotal influence in shaping consumer perceptions and preferences [25]. The EU quality schemes, including PDO, PGI, TSG, and organic certifications, have been instrumental in differentiating products based on their unique qualities and origins. These schemes not only ensure the protection of distinctive products but also establish a framework for producers to have their products recognized and effectively communicate their distinctive qualities to consumers. This process involves stringent rules and regulations laid down by the European Commission, ensuring the integrity and authenticity of the products [26].

The impact of these certifications extends beyond mere labeling. Geographical indications (GIs), for instance, are crucial in marking products that are renowned for their qualities or reputation due to their geographic roots and traditional know-how [27]. For example, PDO products like ‘Brabantse Wal asperges’, a type of white asparagus from the Netherlands, derive their uniqueness from the region’s specific environmental conditions and local expertise [28]. Similarly, PGI products, while less restrictive, maintain a connection with their place of origin, enhancing consumer trust in the product’s authenticity [17]. Organic certification, another significant EU quality scheme, emphasizes the use of organic farming techniques, promoting sustainable agricultural practices. This certification has gained prominence among consumers who are increasingly environmentally conscious and concerned about sustainable food production methods [29].

A comprehensive analysis by the EU-funded Strength2Food project revealed that these quality schemes offer substantial benefits, including new job opportunities, fair pricing for high-quality products, and the preservation of cultural practices. However, challenges remain in maximizing their potential [30]. These include the need for a more streamlined registration process, stronger measures against fraud, and enhanced consumer education on the significance of these labels and their differentiation from conventional products. Currently, the recognition and understanding of these labels among EU consumers are limited, indicating a need for more effective communication strategies to highlight the benefits of these labels [30].

In the evolving landscape of foodstuff economics, the role of food quality certifications, particularly those promoted by the EU, has become increasingly significant. These certifications—PDO, PGI, and TSG—are not just for safeguarding food quality, they also serve as a crucial factor in consumer decision-making processes. The EU’s promotion of these labels aims to safeguard producers of food possessing unique attributes and aid consumers in their choice-making process, highlighting the symbiotic relationship between food quality assurance and consumer trust [31]. A key aspect of these certifications is their contribution to sustainable goals, addressing consumers’ growing concerns regarding food safety, authenticity, and environmental sustainability. These certifications provide a reliable verification of product authenticity and are influential in shaping consumer food choices [32]. The presence of EU labels on agricultural and food products fosters a positive consumer perception, as these labels assure that the product’s origins can be pinpointed to a particular production region and a known process. This transparency is crucial in building consumer trust and confidence in the quality and provenance of their food choices [33].

Recent trends indicate that consumer demand for food quality has significantly elevated. However, it holds significance to note that this interest and the perception of food quality are influenced by various social and demographic factors such as age, income, education, and household structure [34]. This implies that the impact and relevance of EU quality certifications can vary across different consumer segments, necessitating a nuanced understanding of these dynamics in marketing and policymaking [34]. Furthermore, the last few decades have seen a shift in consumer concerns toward healthier lifestyles and environmental care. These concerns are not just passive preferences but active factors influencing changes in food purchasing intentions and perceptions of food quality. The increased awareness and demand for high-quality, environmentally friendly food products have catalyzed changes in the food market, with EU quality certifications playing a central role in this transformation [35].

### 2.3. Role of Certification in Decision-Making

The significance of food certification in consumer choice has gained considerable attention in the last few years. The European Commission’s Joint Research Centre (JRC) and Safe Food Advocacy Europe (SAFE) have highlighted the significant impact of how food information, including certification labels, is communicated to consumers. Scientific studies underscore the need for clarity in food labeling, particularly in areas like nutrition labeling, digital communication, and origin labeling. This clarity is essential for consumer confidence and informed decision-making [36]. Contemporary consumers demand greater transparency in food labels. They seek information about the nutritional content, such as calories, sugar, sodium, or fat levels, and the presence of chemical additives or harmful substances. The demand for transparency extends to understanding the health benefits of the products they consume [37].

The digital realm of food labeling is emerging as an important aspect of consumer decision-making. The JRC acknowledges the need for more research comparing traditional labeling with digital means of providing food information. This indicates a growing trend where consumers are increasingly relying on digital platforms for food information, necessitating a balance between physical labels and digital content to meet consumer needs and expectations [36]. Thus, EU food certifications play a critical role in consumer decision-making. The emphasis on clear, transparent, and easily understandable labeling—both in physical and digital forms—is central to helping consumers make informed choices. As consumer preferences continue to evolve, the importance of these certifications in guiding and influencing consumer behavior is likely to increase, underscoring the need for continuous research and adaptation in food labeling strategies [38,39]. These schemes, PDO, PGI, and TSG, which cover more than 3400 names, including agricultural, fishery, and aquaculture products, wines, and spirit drinks, confer intellectual property rights and ensure the protection of specific products’ names, characteristics, quality attributes, and traditional production methods [11].

By ensuring the authenticity and quality of products, these labels enable consumers to make informed decisions, aligning with their preferences for products with a known origin and traditional production methods. This aspect of consumer decision-making underscores the importance of effective communication and education about these labels, to enhance their recognition and understanding among consumers [25]. The agricultural and food certifications, PDO, PGI, and TSG, significantly influence consumer decision-making by assuring quality, authenticity, and traditional production methods. While these European schemes have been effective in providing benefits to producers and maintaining market integrity, ongoing efforts to enhance consumer awareness and simplify registration processes are essential to fully realize their potential and impact on consumer behavior [40].

A study carried out by [41], spanning six European nations (Belgium, Norway, Poland, Spain, France, Italy) with 4828 participants, revealed varying degrees of awareness and use of these labels. PDO labels were recognized by 68.1% of the participants, significantly higher compared to PGI (36.4%) and TSG (25.2%). Interestingly, awareness was higher in men and individuals aged above 50 years. The primary driver for consumers using these labels was the belief that they signify superior product quality. This quality perception is enhanced by an interest in the product’s origin and quality information provided by the label. The study also highlighted that consumer interest in food origin and support for the local economy are key motivators for choosing products with these labels, though these factors did not directly influence TSG-label use. The findings suggest that efforts to promote consumer interest in origin and quality information through EU quality labels are beneficial [41].

However, other studies indicate that EU quality labeling schemes do not always resonate with consumers [31,42]. It was found that consumer awareness of these labels is generally low, with notable differences between nations. Awareness was higher in southern Europe, possibly due to a higher number of registered products in these regions. From the viewpoint of consumers, food quality encompasses ‘experience qualities’ such as convenience and taste, likewise ‘credence qualities’ including origin, production, and nutritional value. Consumers rely on these ‘quality cues’ in stores, but the effectiveness of EU quality labels depends on the extent to which consumers are aware of, comprehend, and incorporate them into their decision-making [31]. The renewed engagement in traditional foods has led many food producers to leverage these quality schemes as marketing tools. This trend reflects the growing consumer preference for products distinguished by their geography or origin. Consumers are making inferences about traditional origin and production methods through various methods of market communication, indicating a nuanced understanding and preference for products with such labels [5].

### 2.4. Consumer Clustering on EU Quality Schemes Food Products

Exploring further the complex relationship between consumer trends and certification reveals that certification plays a pivotal role in decision-making processes. This role extends beyond merely providing assurance of quality and authenticity to influencing consumer preferences and behaviors. In the realm of EU quality scheme agricultural and food products, understanding consumer clustering becomes crucial [43]. The exploration of various studies on PDO, PGI, and TSG products uncovers diverse consumer clusters, each exhibiting unique characteristics and preferences. These clusters that will be highlighted reveal the multifaceted nature of consumer behavior in the context of certified European food products [44].

In line with these aspects, a study conducted by [26] delves into Italian consumers’ awareness, perception, and awareness of EU quality certifications such as PDO, PGI, TSG, and organic labels. Conducted through a web-based survey with 212 participants, the study used descriptive statistics, factor analysis, and cluster analysis. It revealed increased consumer awareness and consumption of products with these certifications. Notably, a significant portion of respondents recognized PDO, PGI, and organic products, though fewer could identify TSG products. The study [26] also assessed the understanding of the assurances provided by PDO and PGI certifications, highlighting that higher-educated consumers valued these certifications more and supported local economies. These insights have practical implications for market communication strategies of EU-certified food products, both nationally and internationally. Within a study by [45], significant attention is given to the relationship between the consumer and the PGI label. This area of study is of the greatest interest to the scientific community. This cluster mainly focuses on Spanish PGI products, including beef and lamb meat. Other products of interest in the cluster are fruits and vegetables, like melons in Spain, chestnuts in Italy, and lentils in Greece, thus providing insights into consumer preferences and behavior toward these PGI products [45].

The study [6] provides an in-depth analysis of the market potential for Serbian PDO products, specifically focusing on Petrovac sausage and Futog cabbage. The study involves both qualitative and quantitative analyses, including interviews with authorities and producers, as well as consumer surveys. It seeks to understand market perspectives, consumer behavior, and attitudes toward PDO/PGI food products, and their readiness to pay for them. The study identifies different consumer clusters based on their attitudes and willingness to pay for PDO products. These clusters include consumers highly interested in PDO products, moderately interested, and those not willing to pay a higher price for PDO products. This segmentation provides valuable insights into consumer preferences and perceptions regarding PDO-labeled foods in Serbia and can guide marketing and communication strategies for these products [6].

In the results presented by [46] are discovered insights into the awareness and perception of PGI/PDO labels among Romanian consumers. The study [46] utilizes a self-administered questionnaire to explore the notoriety of Romanian GI products and consumer awareness of these labels. The study reveals that most respondents were unfamiliar with the importance of PGI and PDO logos. Between the GI products, PGI “Salam de Sibiu” and PGI “Magiun de Topoloveni” had the highest levels of awareness and consumption. The research highlights the lack of consumer recognition for PDO and PGI logos in Romania and suggests that supermarkets are the primary locations for purchasing GI products, with preferences varying across products. The study [5] goes beyond and investigates consumer choices and willingness to pay for cheese products with quality labels in France and Italy. It focuses on Parmigiano Reggiano and Comté cheeses, combining PDO labels with other quality features like Mountain Product and organic labels. The study [5] uses a random-parameter logit model to analyze online discrete choice experiments and examines consumers’ readiness to pay for these distinct cheese products. It also identifies the impact of personal characteristics on preferences, revealing that price is the most impactful factor, followed closely by the PDO quality label, particularly when paired with a second quality feature. Two consumer clusters in each country with favorable perceptions regarding quality-labeled food products were identified, providing valuable insights for tailored marketing strategies [5].

The authors in [7] focused on identifying citizen profiles based on their socio-economic characteristics and their perception of the primary factors influencing food security. This research, conducted through an online survey in the Metropolitan City of Bari, utilizes K-means cluster analysis. It identifies four distinct citizen clusters: “Law-confident”, “Hedonist”, “Capitalist”, and “Conservatory”. These clusters differ in their confidence in governance, quality certification, and their perceptions of standardization in food production and governance power. The findings can guide policymakers in creating more effective urban food policies and strategies for food security [7]. A more comprehensive study [47] focuses on identifying the impact of various quality indicators on Italian olive oil consumers through a hierarchical cluster analysis. It focuses on understanding Italian consumers’ preferences for olive oil based on different quality signals—organic, local, and PGI labels. Using a conjoint analysis followed by a clusterization approach, the study identifies four distinct consumer groups with varying socio-demographic attributes and hierarchical preferences regarding certification schemes. The research provides insights into the trade-offs consumers consider between these quality signals and how they interpret information from different certifications. This study is valuable for understanding consumer segmentation in the olive oil market and for guiding marketing strategies that align with specific consumer preferences in Italy [47].

A study conducted by [48] explores consumer preferences for the Italian cheese Provolone Valpadana, examining attributes like origin, certification, production system, ‘free from’ labeling, price, and brand. The research employs conjoint analysis to estimate preferences and cluster analysis to identify consumer groups. It reveals that the most preferred characteristic for Italian consumers is the brand, with a preference for lower-priced, organically produced, EU-quality-certified Provolone cheese from Auricchio, not lactose-free. This study provides valuable insights for food companies to better target consumers and promote products effectively. It contributes significantly to the literature on consumer preferences for EU labeling schemes [48]. Focusing on young consumers, [44] investigates young consumers’ perceptions of mountain food products. Using an online survey of 4079 university students, the study employs hierarchical cluster analysis to define four consumer clusters. These clusters show a positive perception of mountain products, associating them with sustainable development, local traditions, specialties, and health. This research [44] offers insights into understanding the characteristics sought by younger generations in mountain food products and contributes to the literature on consumer perceptions in mountain market areas. Cluster one consists of younger respondents, mainly aged 18–21, with a preference for fresh vegetarian mountain food. They are price, packaging, and brand conscious, and view mountain products as a means to consume healthy, tasty, and natural food, believing in their production within mountain areas using local raw materials. Cluster two includes young individuals who favor meat from mountain regions and place a strong emphasis on brand. They associate mountain products with environmental, social, and economic sustainability and a connection to tradition and land. Cluster three is characterized by younger respondents, mainly aged 18–21, influenced by price and packaging. They prefer mountain animal-origin food and fresh products like mushrooms, fruits, and vegetables, valuing mountain products for their certified quality and health benefits. Cluster four involves respondents over 21 years old, affected by origin, but also production place. They prioritize animal-origin and processed mountain products and are willing to pay more for these items, valuing them for their connection to tradition and health benefits [44].

Conclusions drawn from the investigation carried out by [47] identified four main clusters of olive oil consumers: locally, basic, popular, and premium. Each cluster reflects distinct consumer preferences and behaviors. The group of locally produced olive oil consumers prioritizes the healthful and nutritional properties of olive oil and shows a strong preference for locally produced oils. They are typically from smaller households with southern Italian origins and higher education levels. The cluster of basic olive oil consumers is defined by a focus on price and affordability, showing less interest in certification or specific product characteristics. The individuals in the group of popular olive oil consumers tend to favor medium-quality, well-known-brand olive oils. They value health and dietetic-nutritional reasons for consumption, often choosing oils based on affordability and mild sensory attributes. The premium olive oil cluster prefers high-end olive oils, valuing their healthful and nutritional properties, and showing a preference for PDO-labeled oils. They tend to be origin-conscious and appreciate intense sensory characteristics of the product [47].

Results garnered from the research conducted by [49] demonstrate how quality attributes affect consumer perceptions and anticipations of grated Parmigiano Reggiano cheese. Through a survey conducted in hypermarkets, the study identifies four clusters based on consumer preferences for various quality cues and attributes: Cluster one focuses on origin and packaging, while cluster two emphasizes price, brand, and quality certification. Cluster three prioritizes the sensory attributes, and cluster four combines sensory attributes with brand, quality certification, and price. This segmentation provides insights into consumer quality perception, particularly in the context of traditional food products like Parmigiano Reggiano cheese [49]. Outcomes derived from a study carried out by [50] exhibit consumer behavior toward PGI-branded cheese in Turkey. It categorizes consumers into three clusters based on consumption frequency: heavy, medium, and light users. The group of heavy users places high emphasis on intrinsic product characteristics such as food safety, sensorial characteristics, and nutritional value. They are motivated to buy Erzurum Civil cheese based on these intrinsic qualities, particularly appreciating the PDO/PGI cheese’s region of origin. The medium users’ consumers are influenced more by extrinsic product attributes. They value the actual product image and visual attributes, with a focus on the branding of PGI/PDO. Their purchasing decisions are based on these extrinsic factors, emphasizing brand and visual appeal. Like medium users, light users are motivated by extrinsic product attributes. They pay close attention to the product image and visual qualities of the cheese, with an emphasis on branding and presentation. The study [50] suggests that marketing tactics for Erzurum Civil cheese should be tailored to these distinct consumer segments, focusing on intrinsic quality attributes for heavy users and extrinsic qualities for medium and light users. This approach thus enhances consumer satisfaction and demand for each segment [50].

## 3. Materials and Methods

The proposed aim was to evaluate the influence of digital marketing on the behavior of Romanian consumers in choosing Romanian agricultural and food products certified with EU quality schemes. The research methodology relies on the use of a quantitative method, through the sociological survey based on a questionnaire—a systematic method of collecting data from a population to gather insights and understand patterns related to human behavior, preferences, attitudes, and social contexts [51]. This research method is widely employed in the field, as demonstrated by authors from the literature [51,52,53,54]. In the current study, the sociological survey was meticulously designed to ensure comprehensive coverage of participants’ perceptions, experiences, and behaviors related to EU quality scheme agricultural and food products.

### 3.1. Variables Based on Previous Studies

As part of the study’s methodology, the selection of questionnaire variables was informed by an in-depth analysis of previous studies. Thus, the questionnaire was developed around a series of items, grounded on the following concepts from the literature (Table 1)

Quality certifications and brand image significantly influence consumer trust and preference [15,55,56]. The questionnaire examines the impact of these factors on consumer perceptions and purchasing decisions, grounded in literature that explores their effectiveness in fostering a positive product image and consumer loyalty for certified agricultural and food products.

Awareness, perception, and price significantly shape consumer attitudes and behaviors toward certified products. Reflecting extensive research demonstrating these factors’ roles [45,57,58,59,60,61,62], the questionnaire assesses how assisted notoriety (the presentation of logos to the respondents) impacts consumer awareness and alters perceptions of value, also influencing willingness to pay for quality-certified products.

The strategic positioning and advertising of certified products have been shown to significantly impact consumer engagement on EU-certified food products [51,63]. The questionnaire gauges the effectiveness of advertising and product strategies, informed by studies highlighting their importance in driving consumer awareness and purchase intent for certified agri-food products.

The questionnaire explores consumer responses to packaging and labeling, with a particular focus on certification and quality information. This approach investigates how packaging design and the information it conveys can influence perceptions and choices among consumers, enhancing product attractiveness toward certified agricultural and food products [31,64,65].

Online purchasing habits and consumer behavior in digital marketplaces are increasingly relevant [66,67,68,69]. In alignment, the questionnaire delves into the nuances of online shopping habits, seeking to understand the specific ways in which digital marketing efforts resonate with consumers.

### 3.2. Questionnaire Development

The questionnaire (Appendix A) contained 41 primarily closed-ended questions, enabling a structured and rigorous quantitative approach to data analysis. Online purchasing, quality certifications, and price-related questionnaire items make use of a Likert scale validated in scientific articles [55,56,70,71,72,73], ranging from 1 (“To a very small extent”) to 5 (“To a very great extent”) to measure responses.

The questionnaire began with a filter question to distinguish between consumers and non-consumers, with the intention of also identifying the barriers to consuming certified products. For consumers, the first part of the questionnaire aimed at gauging the awareness and recognition of specific quality schemes, PDO, PGI, and TSG. Respondents were asked about their familiarity with these schemes and their capacity to recognize related logos by using binary questions and image recognition items, facilitating an assessment of public awareness and visibility of these certifications.

Subsequent components delved into the perceptions of consumers regarding the online promotion of these quality schemes and their perceived impact on product quality. The questionnaire also explored the extent of trust consumers place in these certified products. A significant section of the questionnaire was dedicated to understanding consumer purchasing behaviors and preferences. This included questions on the frequency and locations of purchasing certified products and the factors influencing these purchasing decisions. The questionnaire probed into the consumers’ reasons for choosing certified products, including taste, health benefits, environmental considerations, and regional origins.

The final section of the questionnaire gathered comprehensive socio-demographic data of respondents, including age, gender, education level, income, occupation, and geographic location. This allowed for a detailed segmentation of responses and a nuanced understanding of how different demographic groups perceive and interact with certified agricultural and food products. A hybrid format of response collection was utilized through the application of online and onsite questionnaires to ensure a comprehensive sample.

### 3.3. Data Gathering

The pretesting of the questionnaire was conducted on a separate sample of 64 individuals, distinct from the main study participants. This pretest allowed for the detection of omissions and the clarification of the intended meaning of the expressions used in the questionnaire. Based on the feedback received during this phase, adjustments were made to the questionnaire to improve its clarity and effectiveness, including rephrasing certain questions for clarity, reordering questions to improve logical flow and respondent engagement, and enhancing the visual layout for better readability.

Following the pretesting stage, the revised questionnaire was then distributed to a new and distinct sample to collect the necessary data for the research. The main survey was conducted from April to December 2022, involving a total of 903 consumers and 244 non-consumers. It is important to note that the pretest sample of 64 individuals was not included in the final analysis to maintain the integrity and the validity of the study findings.

### 3.4. Sample Representativeness

As for the representativeness of the obtained sample, the sample corresponds with the profile of Romania’s population who have access to Information and Communication Technology (ICT), according to the 2022 report from the National Institute of Statistics (INS), [74]. Firstly, in terms of the socio-demographic characteristics of the respondents and Internet usage, the collected profile aligns with that of the general Romanian population with access to ICT. According to the INS report, individuals with access to ICT are predominantly from urban areas, aged between 16 and 54 years, who have completed at least high school and have a job. From this perspective, the research sample is comparable to the target population of the current study. Secondly, regarding the gender of the respondents, the proportions are nearly balanced between men and women in Internet usage, thus reflecting the general trends of Romania’s population, and the fact that the perspectives of both genders are well represented in this research.

Also, the representativeness of the sample is considered adequate as it closely mirrors the population quotas outlined in the Statistical Yearbook of Romania 2022 [75]. The sample selection was convenient, yet efforts were made to ensure a balanced coverage across gender and age groups of respondents, in line with the data from the Romanian Statistical Yearbook 2022. Among the consumers, 48% were men and 52% were women, highlighting a diverse demographic, in line with the data from the Romanian Statistical Yearbook 2022 [75].

As for the geographical distribution of the respondents, according to the INS, the North-West and West regions have the second and third highest percentages of Internet usage [74]. The obtained sample includes participants from all development regions of Romania, predominantly from the North-West, Center, and West development regions. Thus, the research results have national relevance and reflect regional variation in consumer behavior and their perceptions regarding agri-food products certified with quality schemes.

### 3.5. Sample Characteristics

The socio-demographic characteristics of the participants are detailed in the subsequent table (Table 2).

### 3.6. Barriers to Non-Consumption

**Figure 1:**
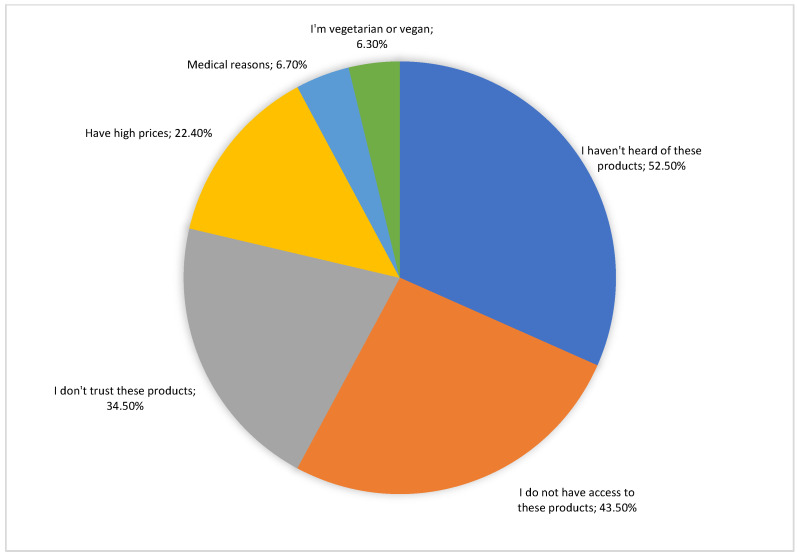
Non-consumption reasons. Source: Own research.

Non-consumers were requested to identify the reasons for not consuming Romanian agricultural and food products certified with EU quality schemes. These barriers to the non-consumption of products certified with such schemes are identified in the literature as being the price [65,76,77], reduced awareness of these certified products due to insufficient promotion at the consumer level [63,78], limited availability [73,79,80], and skepticism [78,81,82]. The reasons for non-consumption are presented in Figure 1. Among the main reasons, it is found that the participants (244 non-consumers) have not heard of Romanian agricultural and food products certified with quality schemes, do not have access to them, do not trust them, and cite high prices.

### 3.7. Data Analysis

Data analysis was conducted using Excel software version 2301, leveraging its capability through the utilization of additional analytical functionalities. To perform the cluster analysis, the first step involved standardizing the variables and entering them into the clustering model. From there, the subsequent action involved removing variables with a *p*-value < 0.05, as they were deemed non-contributory to cluster formation. After their removal, the search for an appropriate number of clusters began. This was achieved by increasing and decreasing the number of clusters tested until a minimal number of iterations in stabilizing the cluster centers was achieved without unduly increasing the total number of clusters. Simultaneously, variables with the lowest F values were eliminated/readded until the model was stabilized after 12 iterations at 4 clusters.

Therefore, those variables that are significant regarding the variation in means and have a substantial F value were retained in the model to provide meaningful insights. The number of cases for each cluster (Table 3) is acceptable, with a ratio of 2.95 between the smallest and largest cluster, among the main 3. A more detailed examination is in the case of cluster 4, which is significantly undersized. Analyzing the elements of this cluster led to the conclusion that it represents a category that should be kept in the analysis because it highlights those cases that, although clear outsiders, manage to maintain a strong core in terms of positioning regarding the theme of the study. Through the analysis of iteration history and changes in cluster centers, it was observed how the data were efficiently grouped into four distinct clusters, with each iteration contributing to a fine-tuning until the final stabilization of the groups. This demonstrates a methodical and iterative clustering process, which is crucial in ensuring the accuracy and relevance of the formed groups.

The final centers of each cluster were calculated as the average for each variable within each cluster. While there are statistically significant differences, socio-demographic factors yield the smallest variation in means when it comes to constructing consumption archetypes of Romanian agri-food products certified with quality schemes. Following the analysis, an optimal number of four clusters was identified based on socioeconomic variables and the degree of consumption, the differentiating elements of these products and decision factors at the time of purchase, online behavior, and general information about the products. The ANOVA test revealed which variables contribute most significantly to the construction of clusters, with those variables having substantial F-values providing the greatest separation between clusters. This step refined the clusters, ensuring they represented distinct segments of consumer behavior toward Romanian agricultural and food products certified with EU quality schemes.

Thus, the cluster analysis comprised a series of tests—standardization of variables, removal of non-contributory variables, adjustment of cluster numbers for stabilization, retention of significant variables, identification of clusters based on socioeconomic factors and consumption patterns, and analysis of changes in cluster centers. These tests were essential for determining the optimal number of clusters [83] and deriving meaningful insights into consumer behavior and consumption patterns.

## 4. Results

As stated previously in the methodology section, the analysis identified four distinct clusters grounded on an integration of socio-demographic determinants and the extent of consumption of Romanian agri-food products accredited with quality schemes. These clusters are further distinguished by several key factors, including the unique attributes of these products, determinants influencing purchasing decisions, online consumer behavior, and general knowledge concerning the products. The clusters have been designated as follows: Eco−Advocates, Les Connaisseurs, Price−Sensitives, and Traditionalists. A comprehensive overview of the primary characteristics attributed to each cluster is presented in Table 4.

The ‘Iteration History’ table (Table 5) denotes the count of each iteration conducted in the clustering process. This reflects the number of steps undertaken to achieve the final configuration of the clusters. The alterations for each iteration indicate how the centers of each cluster have evolved throughout the iterations, an essential aspect in understanding the dynamics of the clustering process.

The values in Table 6 indicate the degree of change in the cluster centers from one iteration to another. A decrease in these values through the iterations suggests that the clustering process has reached a convergence point, where subsequent changes in the cluster centers are minimal.

Following the cluster analysis, the next graphical representations illustrate the findings, the four clusters. The graphs display consumer profiles, consumption patterns, and key variables that define the clusters: Eco−Advocates, Les Connaisseurs, Price−Sensitives, and Traditionalists. These visual aids are designed to provide a clear overview of the data, highlighting the differences and similarities between the clusters. Through these graphics, insights into the consumption archetypes of Romanian agricultural and food products certified with EU quality schemes are presented, facilitating a straightforward interpretation of the results.

**Figure 2:**
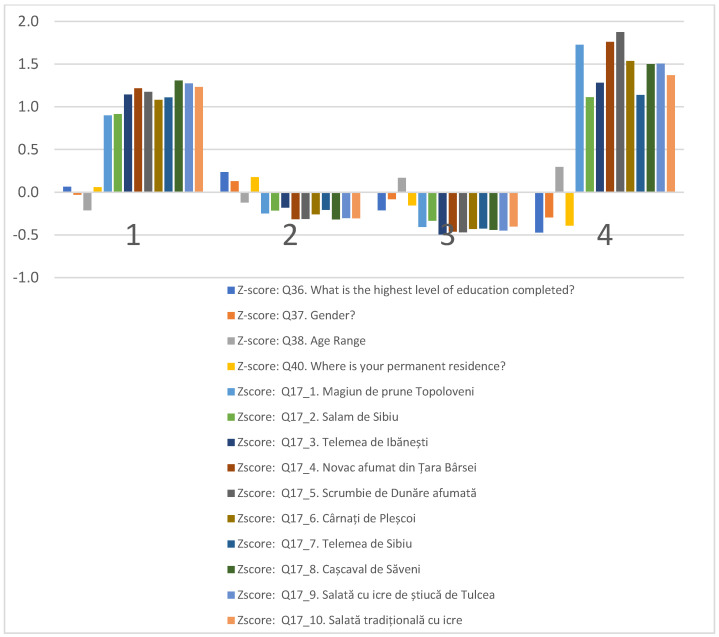
Clustering by socio−demographic factors and the degree of consumption. Source: Own research.

Figure 2 segregates consumers by socio-demographic factors and their consumption frequency of Romanian agri-food products bearing quality certifications. The clusters have been systematically categorized to illustrate the prevailing consumption tendencies alongside demographic attributes of the consumer segments (Figure 2).

Cluster 1, “Eco−Advocates”, is composed of individuals who frequently engage in the consumption of a wide array of Romanian agricultural and food products with quality certifications. This group predominantly encompasses individuals with middle to higher levels of education and includes a balanced mix of males and females, generally younger than those in other clusters.

Cluster 2, “Les Connaisseurs”, consists of occasional consumers of certified products who have attained a higher education level. This cluster has a marginally higher male demographic and is mainly situated in smaller cities or county seats.

Cluster 3, “Price−Sensitives”, is represented by individuals who seldom or never choose certified products. The demographic profile of this cluster is characterized by a lower to middle education background, an older age bracket, and residence in smaller cities or villages.

Cluster 4, “Traditionalists”, encompasses consumers who partake most broadly in food products, notably differentiated from other clusters by their consumption of Scrumbie de Dunăre afumată, Novac afumat din Țara Bârsei, and Magiun de Topoloveni. This cluster is defined by the lowest education level, a higher female presence, and the most advanced age range, predominantly from rural backgrounds.

**Figure 3:**
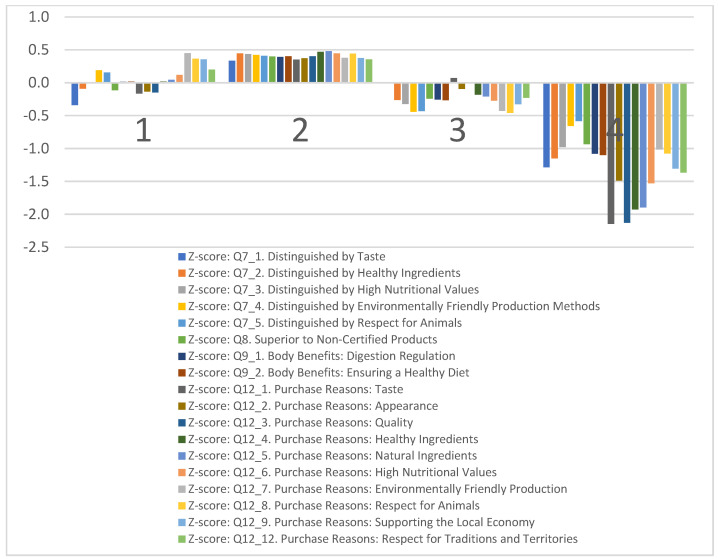
Clustering by differentiating elements of these products and decision factors at the time of purchase. Source: Own research.

The segmentation analysis in Figure 3 within this study focuses on delineating consumer clusters based on the distinctive elements of Romanian agricultural and food products and the decision-making factors at the point of purchase. These clusters are explained by the consumers’ perceptions of product differentiation, particularly in terms of taste, quality, and the various motivations influencing their purchasing decisions.

Cluster 1, “Eco−Advocates”, is made up of those who do not perceive a remarkable difference between certified and non-certified products, with an even lesser extent asserting that taste differentiates them. The main reasons they purchase these products are environmental protection, respect for animals, traditions, and territories, and supporting the local economy.

Cluster 2, “Les Connaisseurs”, achieves high scores for all items, with products being distinctly superior to non-certified ones, and their purchase is motivated by many elements. They seem to be the ones who place the greatest value on what these products have to offer.

Cluster 3, “Price−Sensitives”, consists of those who are not impressed by these products, seemingly attracted by the different tastes of the products. They might be individuals who are either not well acquainted with these products, have not found them available for purchase, have not been curious, or lack sufficient information to understand them. Judging by the size of this group, they also appear to be a target audience for future attempts to expand the customer base.

Cluster 4, “Traditionalists”, seems to see no difference between these products, and they do not purchase them for any particular reasons investigated in the study. They are also those who consume the products but have other purchasing motives.

**Figure 4:**
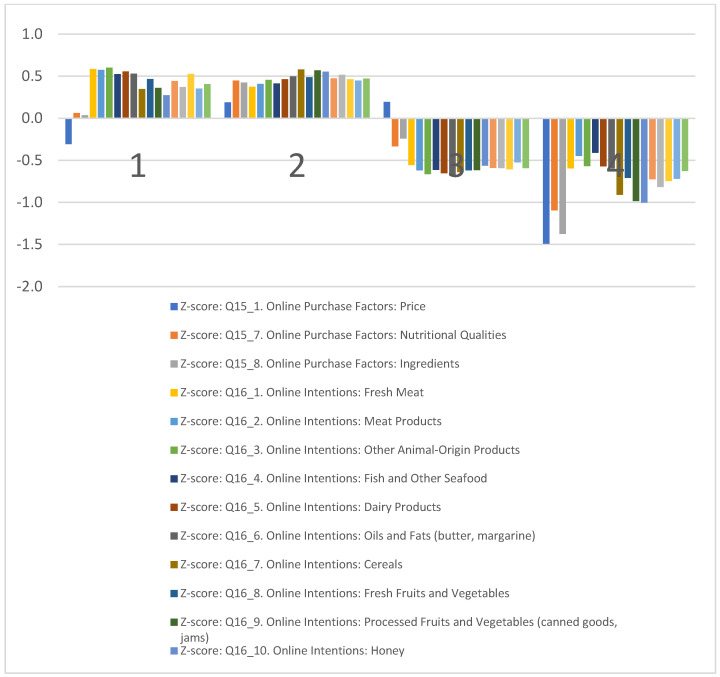
Clustering by online behavior. Source: Own research.

The clustering by online behavior (Figure 4) underscores the varied attitudes toward purchasing Romanian certified agricultural and food products online. The clusters are defined by their preferences regarding nutritional qualities, ingredients, price factors, and their willingness to engage in online purchasing across various product categories.

Cluster 1, “Eco−Advocates”, prioritizes nutritional qualities and ingredients over price when considering the online purchase of certified Romanian products versus conventional ones. They show a readiness to purchase an extensive array of products online, particularly fresh meat, meat products, and other animal-origin products.

Cluster 2, “Les Connaisseurs”, values nutritional qualities, ingredients, and price as crucial factors in their online purchasing decisions. They are also inclined to buy all categories of products online, with a preference for cereals, processed fruits and vegetables, and honey.

Cluster 3, “Price−Sensitives”, is mainly influenced by price in their online purchases, exhibiting a generally lower tendency to purchase all categories of products online.

Cluster 4, “Traditionalists”, seems to have little to no interest in purchasing products online, showing a strong opposition to online shopping. There is a distinct polarization on this issue, with “Eco−Advocates” and “Les Connaisseurs” being more open to online channels, whereas “Price−Sensitives” and “Traditionalists” display resistance.

**Figure 5:**
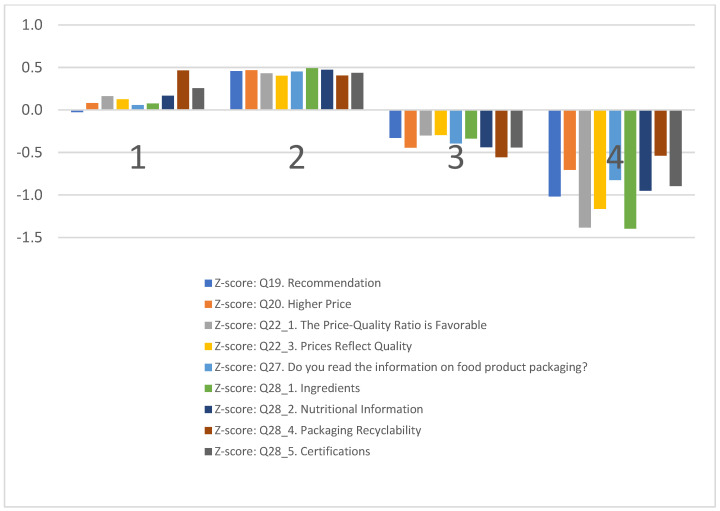
Clustering by general information about the products. Source: Own research.

The clustering based on general information about the products (Figure 5) sheds light on the varying degrees of consumer engagement and perception toward certified Romanian agricultural and food products.

Cluster 1, “Eco−Advocates”, occasionally recommends certified products to others and shows some willingness to pay a higher price. They believe the price/quality ratio is favorable and that the price justifies the quality. While they do read packaging information, their primary interest lies in the recyclability of the packaging.

Cluster 2, “Les Connaisseurs”, strongly values these products, frequently recommending them and showing a willingness to pay extra. They largely agree that the price/quality ratio is favorable and that the price indicates quality. This cluster reads the most information on product labels.

Cluster 3, “Price−Sensitives”, is somewhat less likely to agree with positive statements about the products and rarely reads information on packaging, with the least interest in recyclability.

Cluster 4, “Traditionalists”, is unlikely to recommend the products, unwilling to pay extra, and seldom reads information on packaging, showing the least engagement compared to other clusters.

In this analysis, consumer groups have been clustered according to their attitudes and behaviors toward certified Romanian agricultural and food products, focusing on their propensity to endorse these products, their willingness to invest more in quality assurance, and their interaction with product-related information. Eco−Advocates occasionally advocate for the use of certified products and exhibit a moderate readiness to pay a premium, viewing the price/quality ratio as beneficial and indicative of the product’s quality. Their engagement with packaging information is selective, with a pronounced interest in the sustainability aspect, especially packaging recyclability. Les Connaisseurs demonstrate a strong appreciation for these certified products, often recommending them and expressing readiness to incur additional costs for perceived value. They show a consensus on the favorable price–quality relationship and consider price a marker of quality. This group actively seeks out extensive information from product labels. Price−Sensitives display a cautious agreement with positive assessments of the products and infrequently consult packaging information, showing minimal concern for aspects like recyclability. Traditionalists exhibit a reluctance to recommend certified products, show little to no willingness to pay more for these items, and engage minimally with packaging information, being the least involved among the clusters.

The application of Pearson correlation (Table 7) coefficients has enriched the analysis with depth and statistical rigor by reporting relationships among variables. By establishing a high threshold for these coefficients, the focus was on identifying the strongest and most statistically significant relationships between variables. This methodological approach has revealed the most impactful and meaningful associations within the data, shedding light on the dynamics that influence consumer engagement with certified agri-food products. Among the significant findings, a strong correlation was observed between the preference for purchasing certified animal origin and meat products online (0.897) and the attitudes delineated in the cluster analysis. This association underscores a prevalent consumer preference for certified products in online shopping contexts, reflecting broader trends of increased trust and ethical consideration, notably pronounced among the ‘Eco-Advocates’ and ‘Les Connaisseurs’ clusters. These groups are characterized by their environmental consciousness and discerning purchasing habits, respectively, aligning with the observed correlation.

Furthermore, the correlation coefficient of 0.892 (Table 7), linking healthiness factors and ethical practices in food production, resonates with the values and priorities of the ‘Eco−Advocates’. This trend indicates rising consumer awareness around the impacts of food production processes and a growing demand for transparency and ethical accountability in the agri-food sector. The dataset also reveals a significant inclination across consumer segments to prefer certified products across various categories, suggesting a universal tilt toward a healthier and more ethically conscious lifestyle. This inclination is especially relevant for ‘Les Connaisseurs’, who demonstrate a broad appreciation for the superior quality and ethical standards of certified products.

Lastly, the general appreciation for fair pricing of certified agri-food products indicates a widespread recognition of the value offered by EU quality schemes. This aspect is particularly significant for ‘Price−Sensitives’ and ‘Traditionalists’, who, despite their varied openness to online shopping and certified products, share a concern for price fairness and quality value. These correlations (Table 7), viewed through the lens of the cluster analysis, not only reinforce the distinct characteristics of each consumer group but also highlight overarching consumer trends toward quality and ethics in food consumption.

## 5. Discussion

The exploration of consumer behavior toward EU-quality-scheme-certified agricultural and food products presents a nuanced landscape, where certification not only serves as a marker of quality and authenticity but significantly influences consumer preferences and purchasing decisions. This complex relationship is shown through consumer clustering, both within the context of Romanian consumers—categorized into Eco−Advocates, Les Connaisseurs, Price−Sensitives, and Traditionalists—and broader European consumer studies.

Eco−Advocates, characterized by their environmental and ethical consumption priorities, and Les Connaisseurs, with their discerning appreciation for quality, find parallels in studies across Europe; [26] highlighted increased awareness and consumption of PDO, PGI, and organic products among Italian consumers, particularly those with higher education, mirroring the tendencies of the Eco−Advocates and Les Connaisseurs. This correlation underscores the pivotal role of education in enhancing the recognition and appreciation of certified products. The Eco−Advocates, with their strong environmental attitudes, closely align with the “Law-confident” and “Conservatory” clusters identified by [7]. These clusters share a common trust in governance and quality certifications, underscoring a broader European trend toward environmental and ethical consumption. This alignment suggests that environmental consciousness and ethical considerations are increasingly pivotal in shaping consumer preferences across Europe, not just within Romania. It highlights an opportunity for producers and marketers to further emphasize the environmental and ethical benefits of certified products to appeal to this growing segment [7].

Les Connaisseurs, with their nuanced understanding and appreciation for the quality and authenticity of certified agricultural and food products, find their counterparts in the “Popular” and “Premium” olive oil consumer clusters described by [47]. These groups prioritize healthful and nutritional properties and have a strong preference for products with PDO labels, appreciating their intense sensory characteristics. This similarity indicates a shared valuation of authenticity and quality across different product categories and cultural contexts, reinforcing the importance of clear, transparent labeling and marketing strategies that communicate the unique attributes and origins of certified products.

Moreover, the study by [6] on Serbian PDO products reveals consumer clusters ranging from highly interested to those unwilling to pay more for PDO products. This segmentation aligns with the diversity observed between Eco−Advocates and Price−Sensitives, indicating a commonality in consumer valuation of certification across different cultural contexts. The willingness of Eco−Advocates and Les Connaisseurs to pay a higher price for certified products resonates with the findings of [5], where consumers in France and Italy exhibited similar behavior toward EU-quality-certified cheese products.

The Price−Sensitives and Traditionalists, who exhibit cautiousness toward spending and a preference for traditional purchasing channels, reflect broader consumer segments identified in the literature. The authors in [46] uncovered a lack of familiarity with PDO and PGI logos among Romanian consumers, suggesting an opportunity to enhance awareness and trust through targeted education and marketing, a strategy that could resonate well with the Price−Sensitives and Traditionalists. Similarly, the preference for physical retail experiences over online channels, as indicated by the study’s participants, mirrors the findings of [5], emphasizing the enduring importance of supermarkets and hypermarkets as primary sources for purchasing certified products.

The Price−Sensitives’ cautious approach to spending aligns with the “basic” olive oil consumer cluster identified by [64], where price and affordability are key considerations. This similarity underscores a universal challenge in convincing consumers of the value proposition offered by certified products. It suggests that while certification can signify quality, the perceived cost/benefit ratio is a critical factor influencing purchasing decisions across consumer segments.

The Traditionalists’ preference for purchasing from physical stores mirrors the findings of [5], where consumers favored buying certified agri-food products from farmer’s markets and physical retail outlets over online channels. This preference highlights the enduring importance of physical retail experiences in building consumer trust and loyalty, suggesting that despite the rise of digital marketing, traditional retail channels remain crucial in the consumer journey [5].

The discrepancy between the recognized importance of online information sources and the actual online purchasing behavior presents a critical area for strategic enhancement. This gap, highlighted by the contrast between consumer information-seeking and purchasing behaviors, calls for integrated digital marketing strategies that bridge online engagement with offline purchasing. This challenge is echoed in the study by [7], which identified distinct consumer clusters based on socio-economic variables and perceptions of food security, suggesting that tailored communication and marketing strategies could effectively address diverse consumer needs and preferences [7].

The discussion underscores the necessity of a multifaceted approach in promoting EU-quality-scheme-certified agricultural and food products. By drawing parallels between the Romanian consumer clusters and broader European consumer studies, it becomes evident that while consumer segments may exhibit unique national characteristics, underlying trends and preferences toward certification are largely shared. The findings from the literature, ranging from the studies by [26] and [47], highlight the critical role of education, digital engagement, and tailored marketing in enhancing consumer awareness, trust, and willingness to pay for EU-certified products.

## 6. Conclusions

This research has evaluated the influence of digital marketing on Romanian consumers’ behavior regarding their choice of agricultural and food products certified with EU quality schemes. Through detailed analysis and investigation, several key findings have emerged, directly addressing the outlined objectives and offering insights into the impact of digital marketing in this domain. The study identified four distinct clusters of consumers—Eco−Advocates, Les Connaisseurs, Price−Sensitives, and Traditionalists—each with unique preferences and behaviors influenced by factors such as age, gender, the quality/price ratio, and level of education. Eco−Advocates and Les Connaisseurs showed a higher willingness to engage with and purchase certified products, influenced significantly by digital marketing efforts that highlight the authenticity, quality, and ethical standards of these products.

Digital marketing has played a pivotal role in enhancing consumer awareness about certified agri-food products. The study found that consumers who are more active online, especially Eco−Advocates and Les Connaisseurs, possess a deeper understanding and appreciation of the quality schemes. This underscores the effectiveness of digital platforms in educating consumers and promoting awareness of certification benefits.

The motivations behind purchasing and consuming EU-certified agricultural and food products were strongly linked to digital marketing exposure. Consumers expressed a preference for products that are promoted as healthier, of higher quality, and beneficial for the environment—attributes often highlighted in digital marketing campaigns. Furthermore, the research revealed a significant trust in digital sources of information, making it a crucial channel for influencing consumer decisions.

### 6.1. Theoretical Implications

This study contributes significantly to the academic discourse on consumer behavior and digital marketing within the context of EU-certified agri-food products. By identifying specific consumer clusters and elucidating their distinct behaviors and preferences, this research adds depth to our understanding of the digital marketing environment and its impact on consumer decisions. The cluster segmentation underscores the diversity within consumer responses and refines existing models by integrating digital influence as a core factor in consumer choice dynamic and highlights the pivotal role of digital marketing in enhancing consumer knowledge, trust, and preference for certified products, thereby providing actionable insights for producers, marketers, and policymakers.

Moreover, the study’s findings offer a foundation for developing tailored digital marketing strategies that cater to the nuanced needs of varied consumer segments, promoting a more sustainable, ethical, and health-conscious food consumption pattern. Moreover, they contribute to a broader understanding of digital marketing theories, particularly in how authenticity, ethical standards, and transparency in digital content can effectively sway consumer preferences toward certified agricultural and food products. This research, therefore, extends digital marketing theory by emphasizing the critical role of value-driven content in fostering consumer trust and loyalty.

Through its focus on the Romanian market, this research also opens opportunities for comparative studies across different cultural and geographical contexts, contributing to a global understanding of the dynamics between digital marketing and consumer behavior toward certified agricultural and food products.

### 6.2. Managerial Implications

The implications outlined below are derived directly from the insights gained through this study’s exploration of consumer behavior in response to digital marketing efforts concerning Romanian agricultural and food products certified with quality schemes. These recommendations are intended to guide stakeholders—consumers, producers, and policymakers—toward strategies that leverage the potential of digital marketing to enhance the visibility, appeal, and accessibility of EU-certified agricultural and food products.

As for targeted recommendations, to navigate the complexities of the certified Romanian agricultural and food product market, consumers are advised to seek out information from reliable sources, such as official European Union quality scheme portals and recognized agricultural organizations. This effort toward self-education will not only empower consumers with the knowledge necessary to make informed purchasing decisions but also encourage them to reflect on the wider impacts of their choices on environmental aspects, animal welfare, and local economies.

For producers of EU-certified agricultural and food products, there is a clear imperative to bolster transparency and engage in open communication regarding their production practices and the specifics of their certification standards. Such transparency is crucial in cultivating trust and fostering a sense of loyalty among consumers, particularly those who prioritize ethical considerations in their purchasing decisions. Additionally, producers should harness the potential of digital platforms to connect with their audience, tailoring their online marketing strategies to emphasize the ethical and quality attributes of their offerings, thereby appealing to a digitally engaged consumer segment. For producers and marketers, the present study highlights the efficacy of storytelling about the ethical aspects of production, evidenced by increased engagement among Eco−Advocates and Les Connaisseurs. Utilizing social media analytics to tailor these narratives to the identified consumer clusters can amplify reach and impact, suggesting a targeted approach in digital marketing strategies as pivotal for market penetration and consumer retention.

Policymakers, on the other hand, should be tasked with the development and implementation of initiatives aimed at enhancing public awareness and understanding of the benefits associated with certified EU agricultural and food products. National educational campaigns that elucidate the environmental, health, and economic advantages of these products can play a central role in shaping consumer behavior. Moreover, it is essential for policies to extend support to artisanal and medium-sized producers in their quest for certification, through financial assistance, simplification of certification processes, and marketing aid. Such strategies not only facilitate a broader participation in the certified market but also encourage the adoption of ethical production practices across the agricultural sector. Policymakers are encouraged to leverage the insights from this research by initiating digital platforms that consolidate information on EU-certified products, thereby simplifying consumer access to trusted information. Further, establishing collaborations with digital influencers to educate consumers on the importance of certification can significantly enhance public awareness, aligning policy initiatives with contemporary digital consumption patterns.

By adopting these recommendations, there is an opportunity to foster a more informed consumer base, assist producers in effectively navigating the EU-certified product landscape, and guide policymakers in cultivating a conducive environment for the growth of the certified agri-food product sector. Ultimately, these efforts will contribute to the evolution of a food system that is increasingly sustainable, ethical, and aligned with the health-conscious priorities of contemporary consumers.

### 6.3. Limitations and Suggestions for Future Research Directions

This study offers valuable insights into the role of promoting agricultural and food products certified with EU quality schemes, yet, like any study, it has its limitations. Firstly, the study relies on self-reporting, which may lead to possible distortions of results. Respondents might be inclined to answer in ways that present them in a more favorable light or that better match perceived social norms. Secondly, the results are based on respondents’ subjective perceptions and reports of their purchasing behaviors, which may not accurately reflect actual behavior. Furthermore, this study may not have considered all potential factors that could influence purchasing behavior. This work is focused on the Romanian context, limiting the ability to generalize results to other markets or cultures. For a broader perspective and deeper understanding of the subject, further studies in various geographical and cultural contexts would be necessary. Also, the present study lays the groundwork for future research to explore the effectiveness of various digital marketing strategies across different consumer clusters identified. Subsequent studies could investigate the long-term impact of digital marketing on consumer loyalty toward certified agricultural and food products, including potential shifts in consumer values and behaviors.

## Figures and Tables

**Table 1:** Concepts of the research.

Concepts	References
Brand image	[15,55,56]
Awareness, perception, price	[45,57,58,59,60,61,62]
Advertising	[51,63]
Packaging design	[31,64,65]
Online purchasing	[66,67,68,69]

Source: Own research.

**Table 2:** Socio-demographic characteristics of the respondents.

Socio-Demographic Characteristics	Categories	Frequency	%
Gender	Male	433	48%
	Female	470	52%
Age	18–24 years old	325	36%
	25–34 years old	190	21%
	35–44 years old	135	15%
	45–54 years old	117	13%
	55–64 years old	73	8%
	over 65 years old	63	7%
Level of education	Middle school (8 grades)	36	4%
	Vocational school	45	5%
	High school	352	39%
	Post-secondary school	45	5%
	Higher education (bachelor’s level)	316	35%
	Higher education (master’s, doctoral level)	109	12%
Occupation	Student	280	31%
	Unemployed	9	1%
	Homemaker	27	3%
	Employee	398	44%
	Freelancer	36	4%
	Entrepreneur	81	9%
	Retiree	72	8%
Income	Under RON 4000	542	60%
	Between RON 4001 and 8000	244	27%
	Between RON 8001 and 15,000	81	9%
	Over RON 15,000	36	4%
Residence	In an urban area, county capital	352	39%
	In an urban area, small town	199	22%
	In a rural area, commune/village	352	39%
Development regions—Nomenclature of Territorial Units for Statistics (NUTS)	North-East Development Region	72	8%
	South-East Development Region	27	3%
	South-Muntenia Development Region	36	4%
	South-West Oltenia Development Region	27	3%
	West Development Region	81	9%
	North-West Development Region	416	46%
	Centre Development Region	208	23%
	Bucharest-Ilfov Development Region	36	4%
Medical conditions	Lactose intolerance	27	3%
	Gluten intolerance	27	3%
	Chronic diseases (respiratory, cardiac, digestive, musculoskeletal disorders)	108	12%
	None of the above	741	82%

Source: Own research.

**Table 3:** Cases vs. clusters.

Number of Cases in Each Cluster			
Cluster	1	130	14.40%
	2	383	42.41%
	3	326	36.10%
	4	64	7.09%
Valid		903	
Missing (non-consumers)		244	

Note: The “Missing” category comprises non-consumers and is excluded from the cluster analysis, being only considered in the descriptive analysis (Figure 1). Source: Own research.

**Table 4:** Clusters and related descriptions.

Cluster	Category Description	Frequency	%
Eco−Advocates	This cluster focuses on environmental protection and local economy support, and their interest in packaging recyclability. They also value quality and nutritional properties over price.	130	14.4%
Les Connaisseurs	This cluster has discerning taste and high appreciation for the superiority of certified products. These are individuals who value nutritional quality and are willing to pay extra for it. They are also frequent recommenders of such products.	383	42.4%
Price−Sensitives	This cluster exhibits a price-driven nature and lower frequency of consumption of certified products. They would only buy online influenced by price and seem to be less informed or less interested in the certified products’ specific benefits.	326	36.1%
Traditionalists	This cluster prefers traditional shopping methods, lack of interest in online purchases, and less attention to packaging information. They also have the highest consumption rate for specific food products like smoked fish and preserves but do not seem to care much about the certification.	64	7.1%

Source: Own research.

**Table 5:** Iteration history.

Iteration History				
Iteration	Change in Cluster Centers			
	1	2	3	4
1	9.922	7.51	9.615	8.565
2	1.494	0.409	0.542	0.371
3	0.717	0.233	0.232	0.211
4	0.475	0.155	0.118	0.148
5	0.265	0.115	0.087	0
6	0.071	0.042	0.053	0
7	0	0.07	0.091	0
8	0	0.1	0.136	0.119
9	0	0.047	0.055	0
10	0	0.045	0.053	0
11	0	0.018	0.021	0
12	0	0	0	0

Source: Own research.

**Table 6:** Cluster centers.

Final Cluster Centers				
	1	2	3	4
Zscore: Q36	0.06463	0.23704	−0.21192	−0.47035
Zscore: Q37	−0.03003	0.12959	−0.08225	−0.29555
Zscore: Q38	−0.21029	−0.12089	0.16793	0.29520
Zscore: Q40	0.05828	0.17727	−0.15484	−0.39050
Zscore: Q17_1	0.89922	−0.24734	−0.40685	1.72599
Zscore: Q17_2	0.91560	−0.21322	−0.33292	1.11196
Zscore: Q17_3	1.14400	−0.18142	−0.49457	1.28120
Zscore: Q17_4	1.21704	−0.31474	−0.46091	1.75918
Zscore: Q17_5	1.17406	−0.31345	−0.46815	1.87566
Zscore: Q17_6	1.08171	−0.25828	−0.42957	1.53652
Zscore: Q17_7	1.11119	−0.20677	−0.42360	1.13800
Zscore: Q17_8	1.30686	−0.31918	−0.44078	1.50074
Zscore: Q17_9	1.27281	−0.30193	−0.44849	1.50598
Zscore: Q17_10	1.23123	−0.30512	−0.40154	1.37038
Zscore: Q7_1	−0.34068	0.33586	−0.00644	−1.28504
Zscore: Q7_2	−0.08959	0.44533	−0.26193	−1.14882
Zscore: Q7_3	0.00734	0.43614	−0.32306	−0.97930
Zscore: Q7_4	0.19089	0.42185	−0.44251	−0.65821
Zscore: Q7_5	0.15785	0.40937	−0.42938	−0.58333
Zscore: Q8	−0.11368	0.40069	−0.24183	−0.93511
Zscore: Q9_1	0.01614	0.39209	−0.25498	−1.08042
Zscore: Q9_2	0.01644	0.40440	−0.26578	−1.09967
Zscore: Q12_1	−0.16304	0.35171	0.07283	−2.14459
Zscore: Q12_2	−0.13430	0.37360	−0.09333	−1.48759
Zscore: Q12_3	−0.14750	0.40409	0.00205	−2.12908
Zscore: Q12_4	0.01660	0.46955	−0.18022	−1.92570
Zscore: Q12_5	0.04343	0.47919	−0.20813	−1.89567
Zscore: Q12_6	0.12056	0.44652	−0.27270	−1.52799
Zscore: Q12_7	0.44881	0.38051	−0.42668	−1.01533
Zscore: Q12_8	0.36711	0.44467	−0.45773	−1.07519
Zscore: Q12_9	0.35462	0.37605	−0.32683	−1.30598
Zscore: Q12_12	0.19994	0.35389	−0.22728	−1.36624
Zscore: Q15_1	−0.30917	0.18964	0.19335	−1.49174
Zscore: Q15_7	0.06136	0.44772	−0.33561	−1.09448
Zscore: Q15_8	0.03447	0.42448	−0.24253	−1.37490
Zscore: Q16_1	0.58356	0.37428	−0.55532	−0.59655
Zscore: Q16_2	0.57340	0.40684	−0.61856	−0.44858
Zscore: Q16_3	0.60150	0.45583	−0.66348	−0.57004
Zscore: Q16_4	0.52455	0.41430	−0.61517	−0.41134
Zscore: Q16_5	0.55446	0.46407	−0.65414	−0.57139
Zscore: Q16_6	0.53018	0.49788	−0.67890	−0.59829
Zscore: Q16_7	0.34824	0.57886	−0.64020	−0.91047
Zscore: Q16_8	0.46663	0.48786	−0.61987	−0.70994
Zscore: Q16_9	0.35974	0.56865	−0.61809	−0.98539
Zscore: Q16_10	0.27324	0.55432	−0.56317	−1.00366
Zscore: Q16_11	0.44294	0.47430	−0.59122	−0.72658
Zscore: Q16_12	0.36949	0.51581	−0.59259	−0.81880
Zscore: Q16_13	0.52648	0.46283	−0.60712	−0.74661
Zscore: Q16_14	0.35111	0.44675	−0.52365	−0.71932
Zscore: Q16_16	0.40574	0.47129	−0.59226	−0.62769
Zscore: Q19	−0.02457	0.45690	−0.32740	−1.01665
Zscore: Q20	0.08083	0.46671	−0.44222	−0.70459
Zscore: Q22_1	0.16134	0.43169	−0.30023	−1.38181
Zscore: Q22_3	0.12491	0.40273	−0.29437	−1.16438
Zscore: Q27	0.05705	0.45313	−0.39314	−0.82502
Zscore: Q28_1	0.07687	0.49243	−0.33498	−1.39674
Zscore: Q28_2	0.16753	0.47418	−0.43771	−0.94836
Zscore: Q28_4	0.46540	0.40449	−0.55535	−0.53718
Zscore: Q28_5	0.25680	0.43677	−0.43978	−0.89528

Note: Zscore Q’s represent the standardized variables. Source: Own research.

**Table 7:** Pearson correlations.

Variable Code 1	Variable Code 2	Correlation Coefficient	Statistical Significance
Q16_3	Q16_2	0.897	**
Q12_5	Q12_4	0.892	**
Q16_14	Q16_15	0.830	**
Q16_2	Q16_1	0.827	**
Q25_4	Q25_3	0.824	**
Q12_8	Q12_7	0.820	**
Q22_6	Q22_5	0.802	**
Q16_3	Q16_1	0.786	**
Q17_10	Q17_9	0.785	**
Q15_8	Q15_7	0.783	**
Q25_1	Q25_3	0.779	**
Q25_3	Q25_	0.779	**
Q12_5	Q12_6	0.775	**
Q16_4	Q16_1	0.774	**
Q16_5	Q16_3	0.768	**
Q12_3	Q12_1	0.764	**
Q12_1	Q12_3	0.764	**
Q12_4	Q12_6	0.760	**
Q12_3	Q12_4	0.757	**
Q16_10	Q16_9	0.756	**
Q16_2	Q16_5	0.755	**
Q16_6	Q16_5	0.752	**
Q25_1	Q25_4	0.749	**
Q16_3	Q16_4	0.748	**
Q7_3	Q7_2	0.748	**

Source: ** Statistical significance at the 0.01 level. Note: The table includes Pearson correlations >0.7. The variables represent questions from the questionnaire. Own research.

## Data Availability

The original contributions presented in the study are included in the article, further inquiries can be directed to the corresponding author.

## Appendix A. Questionnaire

(1) Have you heard of the quality schemes used for the certification of agri-food products?
(2) Do you recognize the following logos?
(3) How did you primarily learn about certified Romanian agri-food products?
(4) How much do you think these quality schemes are promoted online among consumers?
(5) To what extent do you consider these schemes an indicator of product quality?
(6) How much do you trust the quality of Romanian agri-food products certified with such schemes?
(7) To what extent do you think certified agri-food products distinguish themselves?
(8) How much do you think certified agri-food products are superior to uncertified products in the same category?
(9) What benefits do you think the consumption of certified Romanian agri-food products has for the human body?
(10) Where do you most often buy certified Romanian agri-food products?
(11) How often do you buy certified Romanian agri-food products online?
(12) How important are the following reasons for you to buy certified Romanian agri-food products?
(13) How much do you trust buying certified Romanian agri-food products online?
(14) When purchasing online, is quality the first aspect you consider?
(15) To what extent would the following factors influence you when buying a certified Romanian product online compared to a conventional one in the same category?
(16) How much would you buy online certified agri-food products in the following categories?
(17) How often do you consume the following certified Romanian agri-food products?
(18) To what extent do your family members consume certified quality scheme agri-food products?
(19) To what extent would you recommend certified Romanian agri-food products to other people?
(20) How willing are you to pay a higher price for certified Romanian agri-food products compared to conventional products in the same category?
(21) What amount would you be willing to pay extra for certified agri-food products compared to conventional ones in the same category?
(22) How do you rate the following aspects of the prices of certified Romanian agri-food products?
(23) How easy was it for you to buy certified quality scheme agri-food products online during the COVID-19 pandemic?
(24) To what extent do you think buying certified quality scheme agri-food products online protected your health during the COVID-19 pandemic compared to buying them in-store?
(25) What are your views on the following statements?
(26) To what extent do you believe certified agri-food products have a positive impact on the environment?
(27) How much do you read the information on food product packaging?
(28) To what extent do you read/view the following information on food product packaging?
(29) How often do you access the following online channels?
(30) How often have you received emails informing you about certified Romanian agri-food products?
(31) Have you seen ads or posts on social media that include certified Romanian agri-food products?
(32) How much do you read/are interested in information received?
(33) To what extent is your diet influenced by?
(34) Do you suffer from any of the following conditions?
(35) What is your net monthly income?
(36) What is the highest level of education you have completed?
(37) Gender?
(38) Age range?
(39) What is your current occupation?
(40) Where is your permanent residence?
(41) In which development region of Romania is your permanent residence?
